# Effects of Tetramethylpyrazine on Functional Recovery and Neuronal Dendritic Plasticity after Experimental Stroke

**DOI:** 10.1155/2015/394926

**Published:** 2015-08-26

**Authors:** Jun-Bin Lin, Chan-Juan Zheng, Xuan Zhang, Juan Chen, Wei-Jing Liao, Qi Wan

**Affiliations:** ^1^Department of Rehabilitation Medicine, Zhongnan Hospital of Wuhan University, Wuhan 430071, China; ^2^Department of Rehabilitation Medicine, Center of Brain Department, Hubei Xinhua Hospital, Wuhan 430015, China; ^3^Department of Physiology, School of Medicine, Wuhan University, Wuhan 430071, China

## Abstract

The 2,3,5,6-tetramethylpyrazine (TMP) has been widely used in the treatment of ischemic stroke by Chinese doctors. Here, we report the effects of TMP on functional recovery and dendritic plasticity after ischemic stroke. A classical model of middle cerebral artery occlusion (MCAO) was established in this study. The rats were assigned into 3 groups: sham group (sham operated rats treated with saline), model group (MCAO rats treated with saline) and TMP group (MCAO rats treated with 20 mg/kg/d TMP). The neurological function test of animals was evaluated using the modified neurological severity score (mNSS) at 3 d, 7 d, and 14 d after MCAO. Animals were euthanized for immunohistochemical labeling to measure MAP-2 levels in the peri-infarct area. Golgi-Cox staining was performed to test effect of TMP on dendritic plasticity at 14 d after MCAO. TMP significantly improved neurological function at 7 d and 14 d after ischemia, increased MAP-2 level at 14 d after ischemia, and enhanced spine density of basilar dendrites. TMP failed to affect the spine density of apical dendrites and the total dendritic length. Data analyses indicate that there was significant negative correlation between mNSS and plasticity measured at 14 d after MCAO. Thus, enhanced dendritic plasticity contributes to TMP-elicited functional recovery after ischemic stroke.

## 1. Introduction

Stroke is the leading cause of long-term disability in the western world, which is a severe disease characterized by its high morbidity, mortality, disability, and recurrence [1]. It has become a heavy burden to patients, families, and societies due to the excessive costs of long hospitalizations, nursing care, and rehabilitation [2]. Ischemic stroke accounts for approximately 87% of stroke [3].

**Figure 1:**
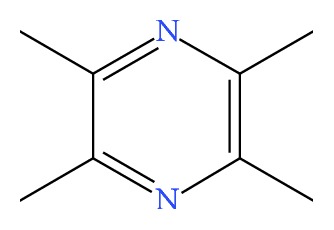
The structure of TMP.

2,3,5,6-Tetramethylpyrazine (TMP, Figure 1) is an active ingredient extracted from a traditional Chinese herbal medicine* Ligusticum chuanxiong Hort*. and has been widely used in ischemic stroke by Chinese doctors [4]. TMP exerts pharmacological effects in multiple ways with multiple targets. TMP is reported to protect ischemia reperfusion injury of heart, brain, and kidney via reducing oxidative stress, attenuating Ca^2+^ overload, inhibiting apoptosis, inhibiting inflammatory reaction, and so forth [5–7]. Besides the above-mentioned effects, it is also demonstrated that TMP can inhibit platelet aggregation, depress blood viscosity, and ameliorate microcirculation [8], which could be another important mechanism to treat cardiovascular and cerebrovascular diseases. Recently, it has been found that TMP could protect hepatic fibrosis by modulating multiple signal pathways [9–11]. Furthermore, TMP had a significant therapeutic effect on diabetic nephropathy [12], which could be mediated by downregulated expression of vascular endothelial growth factor in the kidney and reduction of lipoperoxidation [13, 14]. Additionally, TMP has been reported to have beneficial effects in various types of cancer [15–17]. Specific to ischemic stroke, according to previous studies, TMP can play a protective role through the following mechanisms: antiexcitotoxicity [18], inhibiting inflammatory reaction [19], anti-apoptosis [20], antioxidant activity [21], suppression of calcium [21], thrombolytic effect [22], enhancing neurogenesis, and cell differentiation [23].

There are at least three processes during recovery after stroke: resolution of acute tissue damage, behavioral compensation, and plasticity [24]. Based on the information above, most studies focus on TMP's inhibitory roles in postischemic cascade process in acute phase. However, the effects and mechanisms of TMP on neuroplasticity are still not clear up to now. The plasticity of dendrites is an important component of plasticity [25, 26]. When challenged by ischemic stroke, dendrites in ischemic penumbra (IP) show a series of changes with morphological modifications [27], which suggest that facilitating or optimizing the plasticity of dendrites is likely to be a promising therapeutic target. Indeed, dendritic changes after ischemic injury could be induced by drugs and rehabilitative trainings.

**Figure 2:**
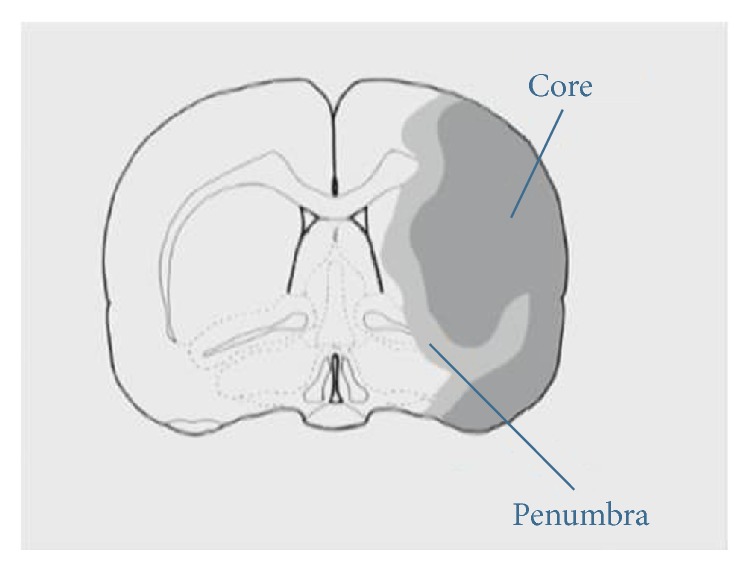
The schematic diagram of ischemic penumbra (IP).

Ischemic penumbra (IP) was first proposed by Astrup et al. in 1981 [28]. It was defined as a region of reduced cerebral blood flow (CBF) with absent, spontaneous, or induced electrical potentials that still maintained ionic homeostasis and transmembrane electrical potentials. It has the potential for functional recovery if local blood flow can be reestablished within a limited period and is a key target for the treatment of acute stroke [29]. It is located in the peri-infarct area and Figure 2 shows schematic diagram of ischemic core and IP.

In this study, we tested the effects of TMP on functional recovery and dendritic plasticity after ischemic stroke. A classical focal cerebral ischemia reperfusion model was induced by middle cerebral artery occlusion (MCAO) in the rat and we conducted a TTC staining. Firstly, we measured the neurological function performance using the modified neurological severity score (mNSS). In order to measure the dendritic plasticity, after behavioral testing, immunohistochemistry was employed to evaluate the levels of microtubule associated protein-2 (MAP-2, marker of neuronal dendrites) and a modified Golgi-Cox staining was conducted to examine dendritic morphologic plasticity. Finally, correlations analyses between functional outcome and plasticity were performed.

## 2. Materials and Methods

### 2.1. Animals

A total of 78 eight-week-old male Sprague Dawley (SD) rats weighing 200–250 g (purchased from Experimental Animal Center of Wuhan University, Wuhan, Hubei, China) were used for this experiment. The rats were acclimated for 3 or more days before the start of any experiments. They were housed in a controlled environment (4 animals per cages, 55 ± 5% relative humidity, 22°C, 12 : 12 h light/dark cycle) and provided with free access to food and water. All experimental procedures involving animals were approved by the Animal Care and Use Committee of Wuhan University Medical School. We made all efforts to minimize the number of animals used and their suffering.

### 2.2. Model

MCAO was induced using the modified intraluminal filament technique [30]. Briefly, rats were anesthetized with 10% chloral hydrate (400 mg/kg) intraperitoneally and, after a median incision of the neck skin, the right carotid artery (CCA), external carotid artery (ECA), and internal carotid artery (ICA) were carefully isolated. The right MCA was occluded with a monofilament nylon filament (Beijing Cinontech Biotech Co., Ltd., Beijing, China) by inserting it through the right CCA and gently advancing into the ICA up to a point approximately 17 mm distal to the bifurcation of the carotid artery. The filament was fixed in place and the animal was allowed to recover from anesthesia. After 2 h, the filament was withdrawn to permit reperfusion. In sham group, all surgical procedures were the same as above without inserting a nylon filament. A heating pad was used to maintain a rectal temperature of 37.0 ± 0.5°C during the surgical procedure.

6 MCAO rats were anesthetized with an overdose of chloral hydrate and sacrificed by decapitation at 3 d after MCAO. The brains were quickly removed and chilled at −20°C for 10 min. 2 mm coronal slices were cut for each brain and immersed in a PBS solution (pH = 7.4) containing 2% triphenyl tetrazolium chloride (TTC) (Sigma, St. Louis, MO, USA) at 37°C in the dark for 30 min. The stained sections were then fixed in 4% paraformaldehyde for 1 h. All stained sections were scanned and the infarct volumes were analyzed by Image Pro Plus 6.0 (Media Cybernetics Inc., Bethesda, MD, USA). To eliminate the effect of brain edema and differential shrinkage resulting from tissue processing, the percentage of infarct volume was calculated as reported previously [31].

### 2.3. Grouping and Administration

**Figure 3:**
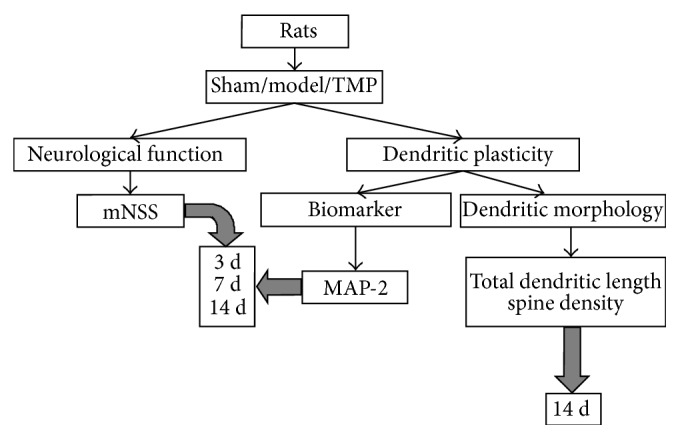
A simple flow-chart of experimental design.

In this study, the animals were randomly assigned into 3 groups: sham group (sham operated rats treated with saline), model group (MCAO rats treated with saline), and TMP group (MCAO rats treated with 20 mg/kg/d TMP (Aladdin Chemistry Co., Ltd., Shanghai, China)). The first administration was conducted immediately after reperfusion. All injections were conducted through intraperitoneal injection daily and in the volume of 5 mL/kg until the day before they were sacrificed. After neurological function test, 54 rats were sacrificed at 3 d, 7 d, and 14 d after MCAO for immunohistochemistry (*n* = 6 in each group at each time point) and 18 rats for Golgi-Cox staining (*n* = 6 in each group) at 14 d after MCAO. A brief flow diagram is shown in Figure 3.

### 2.4. Neurological Function Test

Modified neurological severity score (mNSS) test [32] was measured at 3 d, 7 d, and 14 d after MCAO by an observer blinded to experimental groups. The mNSS is a composite of motor, sensory, reflex, and balance tests and is graded on a scale of 0–18 (normal score 0, maximal deficit score 18). In the severity scores of injury, 1 score point is awarded for the inability to perform the test or for the lack of a tested reflex; thus, the higher the score is, the more severe the injury is. It is classified into three levels: 13 to 18 are graded as severe injury, 7 to 12 as moderate injury, and 1 to 6 as mild injury.

### 2.5. Immunohistochemistry

At 3 d, 7 d, and 14 d after MCAO, rats in each group at each time point (*n* = 6) were anesthetized with an overdose of chloral hydrate and transcardially perfused with 150 mL of 0.9% saline followed by 150 mL of 4% paraformaldehyde. The brains were removed and postfixed in 4% paraformaldehyde overnight. Thereafter, paraffin embedded blocks (bregma: −2 to +2 mm) were obtained and sliced into sections of 6 *μ*m and mounted onto the polylysine-coated slides. Streptavidin-peroxidase (S-P) method [33] was adopted for immunostaining: (1) tissue sections were deparaffinized with xylene and rehydrated in ethanol; (2) they were incubated in endogenous peroxidase blocking solution (Maixin Technology Co., Ltd., Fuzhou, Fujian, China) for 10 min at room temperature; (3) after being incubated with normal rabbit serum (Maixin Technology Co., Ltd., Fuzhou, Fujian, China), the brain sections were incubated overnight with rabbit anti-MAP-2 antibody (1 : 200, Boster, Wuhan, Hubei, China) at 4°C; (4) the sections were incubated with biotin-conjugated second antibody (Maixin Technology Co., Ltd., Fuzhou, Fujian, China) for 15 min; (5) they were incubated with HRP-Streptavidin-Peroxidase (Maixin Technology Co., Ltd., Fuzhou, Fujian, China) for 15 min; (6) the sections were stained with 3, 3′-diaminobenzidine and H_2_O_2_, washed with tap water, and counterstained with hematoxylin. The sections were rinsed with phosphate-buffered saline (PBS, pH = 7.4) 3 times for 3 min between every procedure of staining. Finally, the sections were dehydrated and cover-slipped. To investigate the specificity of the reactions, negative controls were established by replacing the primary antibody with PBS and normal rabbit serum.

For quantitative analysis, three randomly selected sections of each subject and five visual fields (400x) from each section in peri-infarct area were randomly captured under a microscope using a digital camera. Integrated optical density (IOD) was measured using Image Pro Plus 6.0 (Media Cybernetics Inc., Bethesda, MD, USA) for analysis. The analysis procedure was conducted by an investigator in a blind fashion.

### 2.6. Golgi-Cox Staining Procedure

At 14 d after MCAO, rats in each group (*n* = 6) were injected intraperitoneally with a lethal dose of chloral hydrate to induce anesthesia. Remove the brains as soon as possible without perfusion and rinse tissue in double distilled water for 2-3 seconds to remove blood from the surface. Hito Golgi-Cox OptimStain Kit (Hitobiotec Inc., Wilmington, DE, USA) was applied for tissue preparation and staining procedure. The whole Golgi-Cox staining procedure was conducted in strict accordance with the manufacturer's user manual and material safety data sheet. A series of 100 *μ*m thick coronal sections was sliced from the caudal forelimb region of the motor cortex (approximately from bregma to +2.0 mm from bregma) [34] using a microtome (Leica CM1950 cryostat; Leica Biosystems GmbH, Wetzlar, Germany).

### 2.7. Selection Criteria for Pyramidal Cells

To be included for analysis, neurons should be selected according to specific criteria [35]: (1) the dendritic trees had to be well impregnated to facilitate accurate observation and analysis; (2) the cell bodies and dendrites had to be in full view and not obscured by other blood vessels, astrocytes, or clustering of dendrites from other pyramidal cells; (3) they also had to appear intact and visible in the plane of section.

### 2.8. Sholl Analysis

To acquire images for analyzing, layer V pyramidal cells within peri-infarct area were traced at 200x magnification. Pyramidal neurons were readily identified by their characteristic triangular soma-shape, apical dendrites extending toward the pial surface, and numerous dendritic spines [36]. In order to measure the length of dendrites, Sholl analysis [37] was conducted using a Sholl analysis plug-in (available at http://fiji.sc/Sholl_Analysis) for Image J software (National Institutes of Health, Bethesda, MD, USA). The number of intersections of dendrites with a series of concentric rings at 20 *μ*m intervals from the centre of the cell body was counted for each cell. A reflection of total dendritic length can be determined by multiplying the number of intersections by 20 [38]. Five cells per rat were measured for statistical analysis.

### 2.9. Measurement of Spine Density

Dendritic spine density was analyzed from layer V pyramidal neurons within peri-infarct area. For each cell, at least 30 *μ*m long segments of terminal basilar densities (third order or greater, *n* = 5) and apical densities (lower half of the apical segments, *n* = 5) on the same cell were traced at 1000x magnification [39]. The number of spines was counted and the exact length of the dendritic segment was calculated to yield spines/10 *μ*m data [39]. We did not make any attempt to correct for spines hidden by the overlying dendrites. Therefore the data may be likely to underestimate the actual density.

### 2.10. Statistical Analysis

All data was expressed as mean ± standard deviation (SD) and analyzed using SPSS 19.0 software (SPSS Inc., Chicago, IL, USA). Behavior data and immunohistochemical data were analyzed using repeated measures analysis of variance (rANOVA) and when the assumptions of sphericity were violated (Mauchly's test, *P* < 0.05), the Greenhouse-Geisser correction was applied. Post hoc analyses used group designed *t*-test and Turkey's test. One-way analysis of variance (ANOVA) and Tukey's test were used for analyzing dendritic morphological data. Correlations analysis between functional outcome and plasticity were performed using the Spearman correlation coefficients. *P* < 0.05 was considered statistically significant.

## 3. Results

### 3.1. TTC for Model Rats

**Figure 4:**
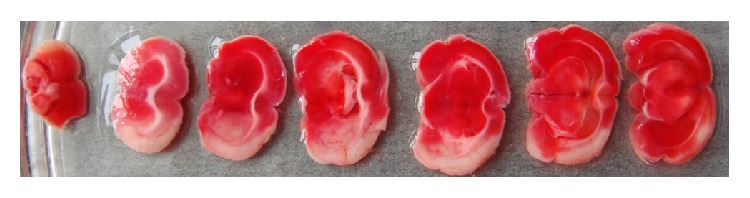
A representative photograph of TTC staining of MCAO rat.

Figure 4 shows a typical photograph of coronal sections of MCAO rat. The infarct region appeared white, and the normal tissue was red. Rats after MCAO exhibited obvious infarction which was located in cortex and striatum. The infarct volume was 38.42 ± 4.42%.

### 3.2. Neurological Functional Assessment

**Figure 5:**
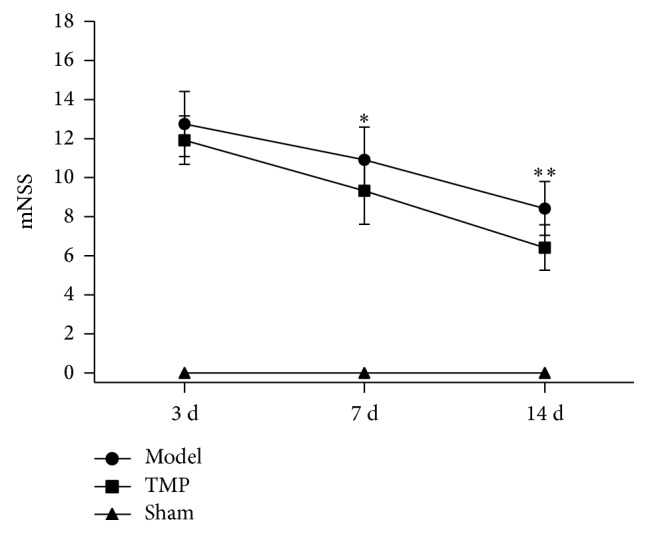
Effect of TMP on neurological status in rats with ischemic cerebral injury. The data were presented as mean ± standard deviation (*n* = 12). ^*^ *P* < 0.05 between model group and TMP group; ^**^ *P* < 0.01 between model group and TMP group.

As shown in Figure 5, for model group and TMP group, rats showed functional improvement with time going on. Repeated measures analysis of variance showed significant group effects (*F* = 11.621, *P* = 0.003). TMP treatment significantly improved functional recovery, as evidenced by improved mNSS at 7 d (model: 10.92 ± 1.68 versus TMP: 9.33 ± 1.72; *t* = 2.281, *P* = 0.033; decreased 14.56%) and 14 d (model: 8.42 ± 1.38 versus TMP: 6.42 ± 1.16; *t* = 3.839, *P* = 0.001; decreased 23.75%) compared with model group. However, there was no significant difference between the two groups at 3 d after MCAO (model: 12.75 ± 1.66 versus TMP: 11.92 ± 1.24; *t* = 1.394, *P* = 0.177). All rats in sham group performed very well without any neurological deficit.

### 3.3. MAP-2 Expression

**Figure 6:**
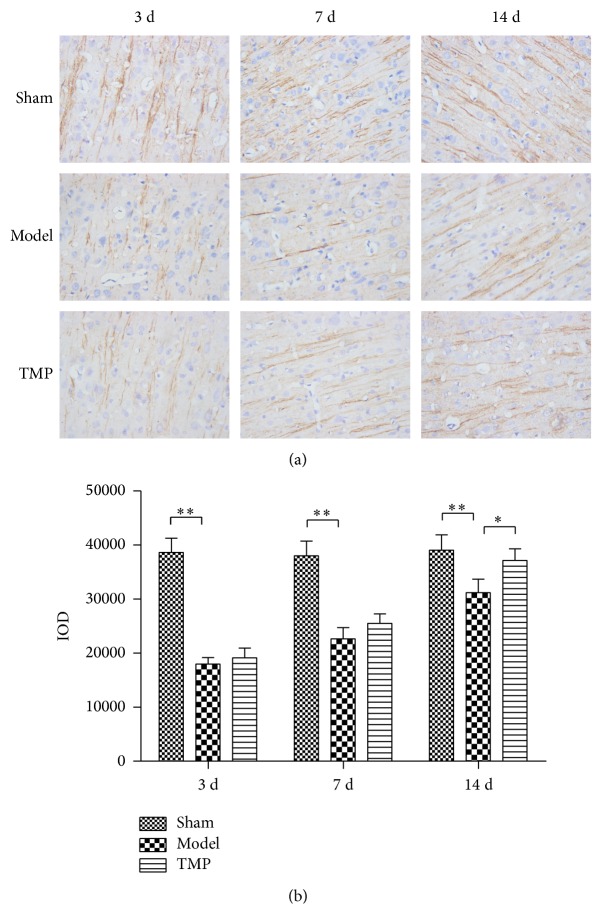
The expression levels of MAP-2 within peri-infarct area of three groups in sham, model, and TMP groups at 3 d, 7 d, and 14 d after MCAO. (a) Immunohistochemical staining of three groups (400x). (b) MAP-2 levels of three groups through measuring the integral optical density (IOD). Data were presented as mean ± standard deviation (*n* = 6). ^*^ *P* < 0.01 and ^**^ *P* < 0.001.

In this study, IOD values were applied to indicate the expression of MAP-2 (Figure 6). In sham group, obvious MAP-2 immunostaining was observed in the dendrites of the cells. Repeated measures analysis of variance showed there was significant group effects (*F* = 77.753, *P* < 0.001). Post hoc analyses showed that there were significant differences between three groups at 3 d (sham: 38635.39 ± 2649.21 versus model: 17958.93 ± 1244.88 versus TMP: 19128.20 ± 1795.69; *F* = 205.913, *P* < 0.001), 7 d (sham: 38009.15 ± 2715.61 versus model: 22635.95 ± 2102.93 versus TMP: 25521.22 ± 1764.14; *F* = 80.61, *P* < 0.001), and 14 d (sham: 39059.86 ± 2831.29 versus model: 31203.85 ± 2478.53 versus TMP: 37147.30 ± 2168.38; *F* = 16.017, *P* < 0.001). Compared to sham group, rats in model group showed significantly lower expression of MAP-2 (3 d, 7 d, and 14 d all *P* < 0.001; decreased 53.52%, 40.45%, and 20.11%, resp.), although they exhibited an increasing trend from 3 d to 14 d after MCAO. TMP treatment resulted in upregulation in MAP-2 expression in peri-infarct area compared to model group at 14 d (*P* = 0.003; increased 19.05%) after MCAO.

### 3.4. Dendritic Morphology

**Figure 7:**
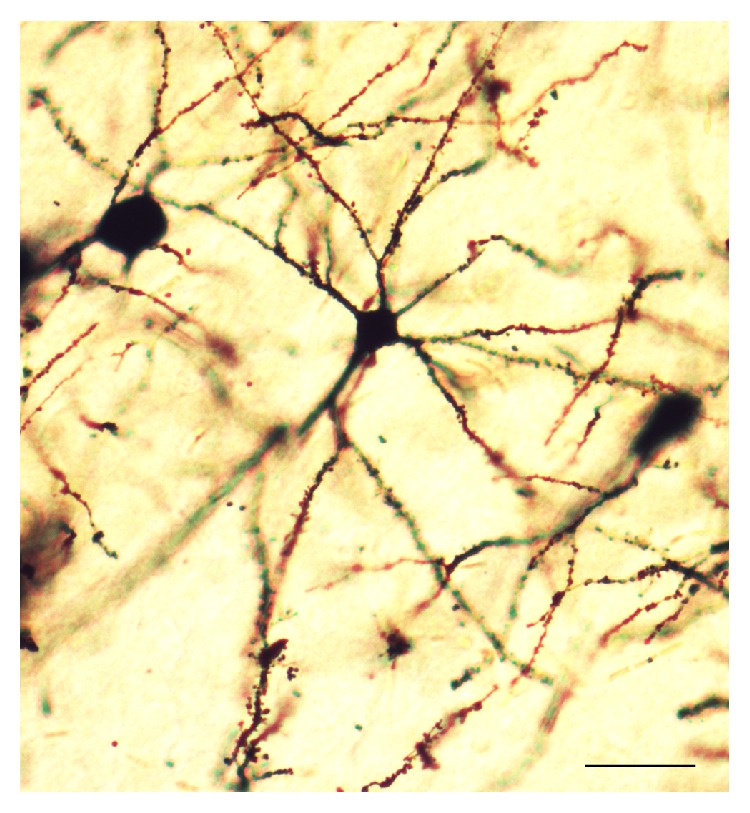
A representative dendritic morphology of layer V pyramidal cells of rats (Golgi-Cox staining). Photomicrograph was viewed at ×200 magnification. Bar = 50 *μ*m.

The morphological analysis presented here is based on a total of 180 neurons from 18 animals. Golgi-Cox staining clearly filled the dendritic shafts (Figure 7) and the spines of neurons from layer V pyramidal neurons. The total dendritic length and dendritic spine density were obtained for analysis.

#### 3.4.1. Total Dendritic Length

**Figure 8:**
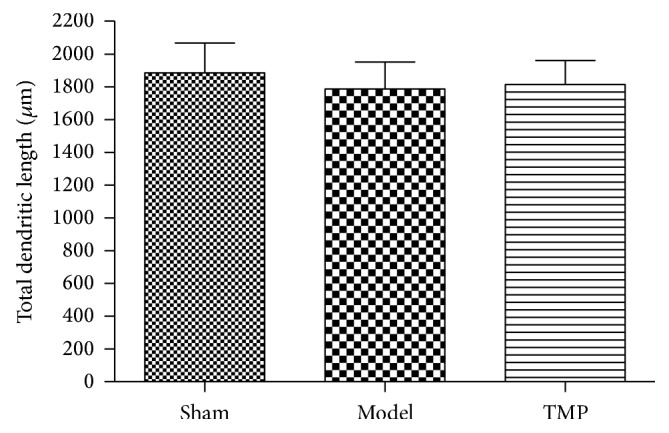
Quantification analysis of effect of TMP on total dendritic length using Sholl analysis. Data were presented as mean ± standard deviation (*n* = 6).

There was no significant difference between three groups at 14 d after MCAO by a one-way ANOVA (sham: 1885.67 ± 180.73 versus model: 1786.00 ± 166.02 versus TMP: 1814.67 ± 145.67; *F* = 0.582, *P* = 0.571) (Figure 8).

#### 3.4.2. Spine Density of Basilar Dendrites

**Figure 9:**
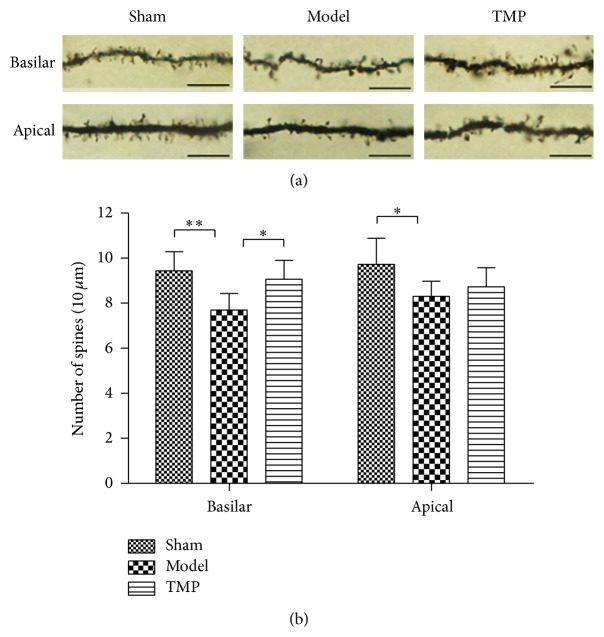
Quantification analyses of effect of TMP on dendritic spine density (basilar dendrites and apical dendrites, resp.). (a) The segments were acquired from layer V pyramidal cells and viewed at ×1000 magnification. Scale bar = 10 *μ*m for all segments. (b) The dendritic spine density was expressed as spines/10 *μ*m and the data were presented as mean ± standard deviation (*n* = 6). ^*^ *P* < 0.05 and ^**^ *P* < 0.01.

For layer V pyramidal neurons, a one-way ANOVA of basilar dendrites spine density found difference between groups at 14 d after MCAO (sham: 9.43 ± 0.85 versus model: 7.70 ± 0.73 versus TMP: 9.07 ± 0.84; *F* = 7.642, *P* = 0.005) (Figure 9). A following Tukey's test revealed that the dendritic spine density in model group was lower than that of sham group (*P* = 0.006; decreased 18.35%) and TMP treatment increased the dendritic spine density compared to model group (*P* = 0.027; increased 17.79%).

#### 3.4.3. Spine Density of Apical Dendrites

For apical dendrites, a similar trend was observed (Figure 9). A one-way ANOVA of spine density also revealed difference between groups at 14 d after MCAO (sham: 9.73 ± 1.16 versus model: 8.30 ± 0.67 versus TMP: 8.73 ± 0.85; *F* = 3.870, *P* = 0.044). A following Tukey's test showed a decrease in spine density of model group compared to sham group (*P* = 0.040; decreased 14.70%), while no significant increase of density was found after TMP treatment (*P* = 0.175).

### 3.5. Correlations Analysis

**Figure 10:**
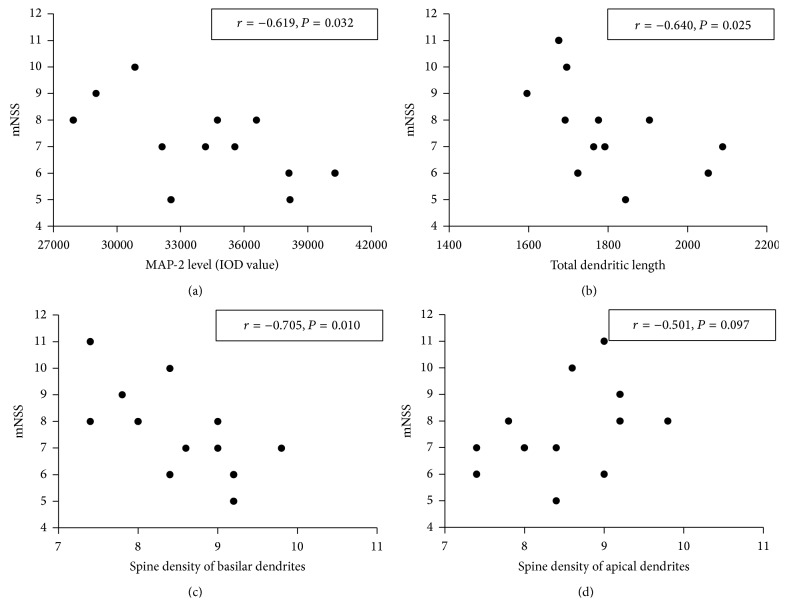
Scatterplots present correlations analysis of mNSS and plasticity measured at 14 d after MCAO. (a) Scatterplots of mNSS and MAP-2 level. (b) Scatterplots of mNSS and total dendritic length. (c) Scatterplots of mNSS and spine density of basilar dendrites. (d) Scatterplots of mNSS and spine density of apical dendrites.

The Spearman correlation coefficients test showed that there were significant negative correlations between mNSS and plasticity measured at 14 d after MCAO (mNSS and MAP-2: *r* = −0.619, *P* = 0.032; mNSS and total dendritic length: *r* = −0.640, *P* = 0.025; mNSS and spine density of basilar dendrites: *r* = −0.705, *P* = 0.010). But there was no significant correlation between mNSS and spine density of apical dendrites (*r* = −0.501, *P* = 0.097) (Figure 10).

## 4. Discussion

MCAO model is classical model and produces obvious infarction induced by focal occlusion of middle cerebral artery [40]. TTC staining is a traditional and widely used method for the research of infarct size. In our study, relatively stable and large-sized infarction in cortex and striatum was induced by MCAO in rats in model group, which showed similar results with previous studies [23, 31].

Ischemic stroke often triggers a complex cascade of cellular and molecular events, including excitotoxicity, calcium overload, oxidative stress, and the following apoptosis and neuroinflammation [2]. TMP could block multiple events of the injury cascade to provide protection [19–21]. Up to now, most studies focused on the inhibitory mechanisms of TMP in the early stage of cerebral ischemia injury and only a few studies analyzed the repair mechanisms of TMP [4, 20, 23]. We reported the TMP's effects on dendritic plasticity in a relative late stage, which may provide a new target and a wider therapeutic window.

In our study, neurological score using mNSS showed obvious difference between sham and model group in all time points, which indicates that MCAO induced relative severe neurological function deficits. There must be a natural recovery process after cerebral ischemia reperfusion injury [41, 42], which could be confirmed by our study. TMP is a small molecular weight medicine and reported to have appreciable blood-brain barrier penetrability [43]. According to our data, TMP could improve functional outcome after focal stroke.

MAP-2 is selectively concentrated in the neuron body and dendrites, which plays a key role in maintaining neuroarchitecture, cellular differentiation, and structural and functional plasticity [30]. MAP-2 has an intimate relationship with ischemic cerebral injury and is considered to be an indication of compensatory dendrites reconstruction in remaining neurons [44, 45]. Several studies revealed that the expression of MAP-2 decreased after ischemic cerebral injury [46–48]. In our study, in sham group, MAP-2(+) cells showed staining mainly in the dendrites of the cells; in ischemic animals, we examined the expression of MAP-2 in peri-infarct area at 3 d, 7 d, and 14 d after MCAO; the level of MAP-2 markedly decreased compared to sham group and persistently increased from 3 d to 14 d after stroke, which was consistent with previous study [48]. These results indicated that the expression of MAP-2 showed a dynamic process after stroke (decreasing in early stage and increasing gradually), which may represent degeneration and reconstruction of dendritic structure. Two studies [25, 49] declared there were a peak point and following downtrend during dendrites reconstruction. However, we did not observe this process which may be due to the relatively short period of observation.

Our data showed that treatment of TMP significantly increased MAP-2 expression level in peri-infarct area after stroke and the neurological function was improved meanwhile, indicating that promotion of the reconstruction of dendrites may contribute to the improvements of neurological function. The mechanism is not clear but may be associated with inhibition of calpains. Calpains could be activated by elevated levels of intracellular calcium after ischemic injury [50, 51], causing proteolysis of numerous neuronal cytoskeletal and regulatory proteins. The increase in calpain expression in the ischemic area was accompanied by a loss of its substrate MAP-2 [52]. TMP is a calcium antagonist and could markedly reverse the increased intercellular free calcium concentration [21]. This effect may contribute to upregulation of MAP-2 level. Correlation analysis showed that there was a significant negative correlation between mNSS and expression of MAP-2, indicating that TMP's effect on improvement of neurological function may be the association with upregulation of MAP-2.

MAP-2 is an indirect marker which can be used for representing dendritic plasticity. However, morphological study is more distinct and more direct for assessments of dendrites. Golgi-Cox staining method has been used broadly for studying morphology of neurites, including quantitative analysis of dendritic length, arborization, and spine density [53], of which spine density is the most important parameter. Dendritic length reflected the total space for synapses and spine density represented the density of excitatory synapses to some extent [54]. Sholl analysis was a classical method for measuring dendritic length, which is an important parameter reflecting dendritic plasticity. We found that the dendritic length of layer V pyramidal cells within peri-infarct area did not change compared to sham group. In fact, the evidence about changes of dendritic length after stroke is controversial; some studies found a shortening of dendrites after cortical lesions [38, 55]; another study found no difference or extension of dendrites in peri-infarct cortex after MCAO [56]. Such paradoxical results are perhaps associated with the absence of a peri-infarct baseline or absence of dynamic study. Brown et al. [57] conducted a longitudinal study and found there was a balance between dendrites extension and retraction after stroke, which may be a mechanism to explain our results. In addition, no obvious alternations of total dendritic length were observed after being treated by TMP, indicating that TMP may fail to affect dendritic length totally at 14 d after stroke. Increasing of dendritic length is good for recovery of stroke, but the result is not good in this regard.

Dendrites and dentritic spines are the primary postsynaptic targets, which receive the majority of excitatory synapses [58]. Previous studies have shown that spine density could be enhanced by drugs [39] or rehabilitative training [59] after experimental stroke, which was likely to play a key role in mediating functional changes that occurred during and after stroke [27]. In our studies, the dentritic spine density of layer V pyramidal neurons decreased significantly in peri-infarct area at 14 d after MCAO, indicating the degeneration of dendrites, which is in accordance with previous study [60]. After chronic treatment with TMP, the spine density of basilar dendrites increased compared to model group; for apical dendrites, there was no significant difference between model group and TMP group. One explanation is that the modifications of basilar dendrites and apical dendrites did not occur at the same time in the recovery period [61]. The degeneration and reorganization of dendritic spines is a complicated process and could be regulated through multiple mechanisms including receptors, scaffolding proteins, and regulators of the cytoskeleton [62, 63]. However, the physiological mechanism responsible for TMP stimulating this increase is unclear in this experiment. Correlation analysis showed that there was a significant negative correlation between mNSS and spine density of basilar dendrites, indicating that TMP's effect on improvement of neurological function may be also the association with increase of spine density of basilar dendrites.

There is a dynamic change of dendrites and dendritic spine after ischemic injury over time [27]. We did not measure the dendritic morphology of other time points, so it is one of limitations that we could not reveal morphological changes during ischemic stroke and recovery.

## 5. Conclusion

TMP may increase MAP-2 level after cerebral ischemia reperfusion and decrease the alterations of neuronal dendritic spines induced by ischemia, suggesting that TMP may have a potential and specific effect on the neuronal dendritic plasticity in rats with transient focal cerebral ischemia reperfusion. Meanwhile, TMP also improved functional outcome after stroke. Taken together, after cerebral ischemia reperfusion, dendritic plasticity is one of the mechanisms that contributed to functional recovery, which might be regulated by TMP.
